# Individual Responses to Repeated Dosing with Anthocyanin-Rich New Zealand Blackcurrant Extract During High-Intensity Intermittent Treadmill Running in Active Males

**DOI:** 10.3390/nu16244253

**Published:** 2024-12-10

**Authors:** Ian C. Perkins, Sam D. Blacker, Mark E. T. Willems

**Affiliations:** 1 Institute of Education and Social Sciences, University of Chichester, College Lane, Chichester PO19 6PE, UK; i.perkins@chi.ac.uk; 2 Institute of Applied Sciences, University of Chichester, College Lane, Chichester PO19 6PE, UK; s.blacker@chi.ac.uk

**Keywords:** anthocyanins, high-intensity exercise, blackcurrant, sports nutrition

## Abstract

Intake of New Zealand blackcurrant (NZBC) extract for 7 days has been shown to improve high-intensity intermittent running (HIIR) performance. Objectives: We examined the repeat response of NZBC extract on HIIR performance. Methods: Sixteen active males (age: 23 ± 3 yrs, height: 179 ± 5 cm, mass: 79 ± 11 kg, $\dot{V}$O_2max_: 55.3 ± 5 mL∙kg^−1^∙min^−1^, velocity at $\dot{V}$O_2max_: 17.2 ± 0.8 km∙h^−1^, mean ± SD) participated. Familiarized subjects completed the HIIR test at individualized exercise intensities with stages consisting of six 19 s high-intensity running bouts interspersed by 15 s of low-intensity running and 1 min of inter-stage rest. The test was repeated at increasing speeds until exhaustion, under four conditions; two with a daily dose of 600 mg of NZBC extract (CurraNZ™, providing 210 mg anthocyanins) and two with a placebo, each over 7 days. The study used a double-blind, randomized, cross-over design with a wash-out period of at least 14 days. Results: For the cohort, there were no differences between the placebo and NZBC conditions for mean heart rate (*p* = 0.071), mean oxygen uptake (*p* = 0.713), and mean lactate (*p* = 0.121) at exhaustion for the HIIR. The NZBC extract increased the mean total running distance and mean high-intensity running distance by 7.9% and 8.0% compared to the placebo. With NZBC extract, 8 of the 16 participants (50%) enhanced in both trials beyond the smallest worthwhile change for total running distance (≥173 m) and high-intensity running distance (≥111 m). For repeated responders, total running distance and high-intensity running distance was increased by 16.7% (95% CI [11.0, 22.4%] and 16.6% (95% CI [11.0, 22.2%]. Three participants had enhanced running performance in one trial beyond the SWC, and five participants were considered non-responders. Conclusions: This is the first study on the repeated response by an anthocyanin-rich supplement on high-intensity running performance. New Zealand blackcurrant extract can substantially enhance intermittent high-intensity running performance in consistent responders. Future work should examine dosing strategies of New Zealand blackcurrant, and whether a repeated response rate exceeding 50% can be attained. These findings suggest that NZBC extract could be beneficial for athletes participating in high-intensity team sports.

## 1. Introduction

In sport and exercise nutrition, supplementation with anthocyanins, such as New Zealand blackcurrant (NZBC) extract, has been found to be an effective nutritional ergogenic aid [1]. NZBC extract supplementation improved exercise performance in 16.1 km [2] and repeated 4 km time-trial cycling [3], enhanced exercise capacity for high-intensity intermittent running [4], reduced slowing of running maximal sprints [5] and improved sports climbing performance tasks [6]. The mechanisms for the performance-enhancing effects of NZBC extract may be attributed to enhanced vasodilation [7], muscle oxygenation [8] and alterations in substrate oxidation [2,9]. However, as is the case in many sports supplement studies, the beneficial effects in studies with NZBC extract have focused mainly on the mean efficacy of supplementation in the cohort.

In keeping with calls to report individual responses to sports supplements [10], some studies have highlighted inter-individual variations in response to NZBC extract, with ~70–90% of participants demonstrating a positive response for cardiovascular and cycling performance measures [2,3], and ~50–70% demonstrating a beneficial response in running-based protocols [4,5,11]. Moreover, Braakhuis et al. [12] reported that the variations in response to anthocyanin-rich blackcurrant supplementation may be attributed to training status, while Willems et al. [13] indicated that the predominant muscle-fiber type may influence the response to NZBC extract.

While inter-individual differences have gained attention, intra-individual variations are often ignored [14,15]. Responses to caffeine ingestion appear to be highly repeatable [16], yet intra-individual responses to sodium bicarbonate supplementation can be inconsistent [17]. It is possible that an individual who does not appear to respond to an intervention in a single observation may do so on another occasion following an identical intervention [18]. Thus, it has been suggested that intra-individual variability should be assessed using repeat period cross-over designs, in which each intervention is repeated at least once for each subject, interspersed by adequate washout periods [19,20]. While such designs may only sometimes be feasible, due to the length of intervention and/or length of washout required, this approach would help clarify an individual’s response consistency [21]. Such steps are suggested for individuals before using ergogenic nutritional supplements in competitive sports settings [22].

Therefore, the present study examined the reproducibility of physiological responses and the performance of high-intensity intermittent treadmill running to exhaustion following NZBC extract supplementation. The primary hypothesis was that NZBC extract intake would enhance high-intensity intermittent running capacity, measured by total running distance covered and distance covered during high-intensity running, but inter-individual variability would exist. Secondly, it was hypothesized that there would be a high degree of reproducibility in response to NZBC extract on an intra-individual basis.

## 2. Materials and Methods

### 2.1. Participants

Sixteen healthy Caucasian men were recruited (mean ± SD, age: 23 ± 3 years, height: 179 ± 5 cm, mass: 79 ± 11 kg, $\dot{V}$O_2max_: 55.3 ± 5.0 mL·kg^−1^·min^−1^, _v_$\dot{V}$O_2max_: 17.2 ± 0.8 km·h^−1^). Participants were recreationally active and participated in sports or exercise with high-intensity and intermittent running. Participants were non-smokers, had no known allergy to anthocyanins, and were instructed not to take additional supplementation during the study. Participants did not receive payment and provided informed written consent. The study was approved by the University of Chichester Research Ethics Committee (approval code: 2425_12, approval date: 21 June 2016) and conformed to the Declaration of Helsinki.

### 2.2. Experimental Design

The study comprised 5 visits over 12–16 weeks. In the first visit, participants performed a rapid ramp treadmill test to exhaustion to determine $\dot{V}$O_2max_. This was followed by a verification phase to confirm $\dot{V}$O_2max_ [23]. Then, participants had a full familiarization of the high-intensity, intermittent treadmill-based running test. For the experimental visits, participants were assigned to the different sequences of four trials, two NZBC extract and two placebo (PL), in a replicated double-blinded, completely randomized cross-over design (Table 1). During the experimental trials (i.e., testing sessions 2–5), participants performed a continuous/intermittent warm-up protocol before completing the high-intensity intermittent running test. The experimental visits were separated by at least 21 days and not more than 45 days, allowing a 14-day wash-out period prior to commencing the next supplementation period of 7 days [24]. In addition, Alvarez-Suarez et al. [25] reported that for an anthocyanin intake higher than the present study, a washout of 15 days returned biochemical and biomarkers of antioxidant status back to baseline.

All the sessions were conducted in ambient conditions (17–19 °C and 60–75% humidity) in an exercise physiology laboratory. The running test was carried out on a motorized treadmill (H/P/COSMOS, Groningen, The Netherlands), set at a 1% gradient. A breath-by-breath gas analyzer (Jaegar Oxycon Pro, Cardinal Health, Basingstoke, UK) was used for expired air collection and analysis. At the start of each session, the system was calibrated with gases of known concentration, and the tube flowmeter was calibrated with a 3 L syringe. The blood samples were analyzed for lactate concentration within 30 s of collection (YSI 2300, Analytical Technologies, Farnborough, Hants, UK). Participants were instructed to record food intake and physical activity in the 48 h preceding the first experimental visit. For subsequent visits, participants were requested to replicate this in the 48 h preceding the visit. No caffeine and alcohol was allowed 24 h preceding each session and participants had to abstain from vigorous exercise. Experimental trials were conducted at the same time in the morning (±2 h), to limit circadian rhythm variation.

### 2.3. Experimental Procedures

#### 2.3.1. Rapid Ramp $\dot{V}$O_2max_ Verification Test

The treadmill test started at an individually determined speed and increased by 0.1 km·h^−1^ every 5 s until exhaustion. $\dot{V}$O_2max_ was recorded as the highest 15-breath average value attained before exhaustion. After a 10 min passive recovery, a verification square-wave test to exhaustion was conducted. The running speeds for the verification protocol were determined by the speed achieved at $\dot{V}$O_2max_ (100% _v_$\dot{V}$O_2max_) by the rapid ramp protocol. The verification square-wave test commenced with 3 min at 50% _v_$\dot{V}$O_2max_, before an abrupt increase to 100% _v_$\dot{V}$O_2max_. Participants were not provided with temporal feedback, but were verbally encouraged to continue during both tests until volitional exhaustion. A consistent peak $\dot{V}$O_2max_ confirmed the attainment of a true $\dot{V}$O_2max_ in the rapid ramp and verification protocols [23].

#### 2.3.2. High-Intensity Intermittent Running Test

Warm-up consisted of a 5 min running stage at 50% _v_$\dot{V}$O_2max_ followed by a 3 min interval consisting of alternating walking at 30% of _v_$\dot{V}$O_2max_ and running at 60% of _v_$\dot{V}$O_2max_, with the transitions occurring every 15 s. Following the warm-up, participants had five minutes for self-selected stretching.

Perkins et al. [4] previously used the running protocol, which involved three phases and was adapted from the Intermittent High-Intensity test developed at Nanyang Technological University, Singapore [26]. The first phase consisted of 5 min running at 60% _v_$\dot{V}$O_2max_. The second phase comprised 7 stages, with each stage lasting a total of 204 s (six repeated high-intensity running bouts lasting 19 s interspersed with active recovery bouts [at 50% _v_$\dot{V}$O_2max_] lasting 15 s) and interspersed with 60 s of passive recovery. A fingertip blood sample was collected for lactate during the 60 s of passive recovery. The speed for the high-intensity running bouts was calculated by a percentage of _v_$\dot{V}$O_2max_, with stage one being set at 80% _v_$\dot{V}$O_2max_. The running speed of the high-intensity running bouts in each stage was then increased by 5% _v_$\dot{V}$O_2max_ (0.9 ± 0.0 km·h^−1^) per stage, up to 110% _v_$\dot{V}$O_2max_ (stage 6). Thereafter, in phase three (≥stage 7), the speed increased by 2.5% _v_$\dot{V}$O_2max_ (0.4 ± 0.0 km·h^−1^) per stage until volitional exhaustion. Note that exhaustion could also be reached during the second phase. The treadmill required ~2 to 4 s for acceleration and deceleration between speeds and to reach the set velocity. The high-intensity running bout speed for stage one was 13.8 ± 0.6 km·h^−1^ and active recovery speed was 8.6 ± 0.4 km·h^−1^. During the test, participants were informed of the beginning and end of a high-intensity running bout, but not the bout or stage number, and received verbal encouragement to perform at maximum effort in all testing sessions. On reaching voluntary exhaustion, the treadmill test allowed the measurement of total distance covered and the distance covered during the high-intensity running bouts. During the treadmill running test, expired air was collected via online breath-by-breath analysis (Jaegar Oxycon Pro, Cardinal Health, Basingstoke, UK). Heart rate (Consultancy RS800, Polar Electro UK Ltd., Warwick, UK) was recorded during each exercise protocol. Immediately upon completion of the treadmill running test, a fingertip blood sample was collected.

#### 2.3.3. Supplementation

Participants were randomly allocated to receive seven days of NZBC extract supplementation [210 mg anthocyanin (delphinidin-3-rutinoside 35–50%, delphinidin-3-glucoside 5–20%, cyanidin-3-rutinoside 30–45%, cyanidin-3-glucoside 3–10%)] per dose of 600 mg CurraNZ™, administered as two capsules per day, CurraNZ™, Health Currancy Ltd., Surrey, UK) on two occasions, or identical-looking PL (600 mg microcrystalline cellulose M102, administered as two capsules per day) on two occasions (Table 1). Previous studies on dose–response effects of intake of New Zealand blackcurrant seem to suggest more beneficial metabolic, cardiovascular and endurance performance responses overall with intake of 600 mg of extract compared to 300 mg [27,28,29,30]. The capsules in the present study were provided by a researcher not involved in the practical testing. On the morning of the final day of supplementation, subjects consumed their last supplement two hours before testing. Participants were also asked to arrive fully hydrated and consume a slice of toast without butter ≥ 2 h before each testing session.

### 2.4. Data Analysis

#### Oxygen Uptake

Breath-by-breath oxygen uptake ($\dot{V}$O_2_) from the running test was time-aligned by the start of the test. Data were subsequently examined to exclude errant breaths, and values more than four standard deviations from the local mean were removed. End $\dot{V}$O_2_ was calculated as the highest 15-breath average value attained prior to exhaustion during the final attempted bout.

### 2.5. Sample Size

The required sample size was not calculated, but was based on a previous study examining the effects of NZBC extract on HIIR capacity that had a sample size of 13 [4]. A sample size of 16 participants completed all sessions.

### 2.6. Statistical Analysis

Trail sequence allocation was revealed on completion of all data analysis. Statistical procedures were conducted using GraphPad Prism version 5.00 for Windows (GraphPad Software, San Diego, CA, USA). Significance was accepted at *p* < 0.05. The cohort responses for heart rate, oxygen uptake, and lactate were analyzed (one-way ANOVA and post hoc tests) for stage 3 in the second phase of the test, because all participants completed stage 3 in all conditions. Cohort responses for heart rate, oxygen uptake, lactate and exercise performance were also analyzed at exhaustion for the high-intensity intermittent running test. One-way ANOVA and post-hoc tests analyzed trial order effects.

To assess the inter- and intra-individual variation in the running performance response to NZBC extract, two analytical approaches were used:

1. For the inter-individual responses for running performance, the first and second NZBC extract trials for the participants were paired to their first and second PL trials [17]. Pearson’s correlation coefficients were then calculated for the differences between the paired NZBC extract and placebo trials [31]. Thresholds of 0.1, 0.3, and 0.5 were used to label correlation coefficients as small, moderate, and large, respectively [32].
2. For the intra-individual responses for running performance, a smallest worthwhile change (SWC)-based threshold was used to assess meaningful individual responses [15]. Since there were no differences for the total running distance and high-intensity running distance in the placebo conditions (paired *t*-tests, *p* = 0.353 and *p* = 0.254), the data were pooled, and the standard deviation was used to calculate the SWC using Cohen’s d effect size (ES) (i.e., 0.2 × SD of the placebo data). Intra-individual responses were determined by counts of responses with intake of New Zealand blackcurrant beyond the SWC. Participants with two distances higher than the SWC were repeated responders, participants with one were occasional responders, and participants with none were non-responders to intake of anthocyanin-rich New Zealand blackcurrant.

## 3. Results

### 3.1. Heart Rate, Oxygen Uptake, and Lactate Responses of Stage 3 in the High-Intensity Intermittent Running Test

All participants completed the four experimental sessions (i.e., 2× placebo and 2× NZBC extract) with three stages of the high-intensity intermittent running test (i.e., completing 18 bouts of 19 s running) before reaching exhaustion in stage 4 and beyond. Table 2 provides the cohort observations for the heart rate, oxygen uptake, and lactate for stage 3 of the high-intensity intermittent running test. For the cohort, there were no differences in heart rate (*p* = 0.778), oxygen uptake (*p* = 0.974) and lactate (*p* = 0.127) for stage 3 of the high-intensity intermittent running test.

### 3.2. Heart Rate, Oxygen Uptake, and Lactate Responses at Exhaustion During the High-Intensity Intermittent Running Test

Table 3 provides the cohort observations for the heart rate, oxygen uptake, and lactate at exhaustion during the high-intensity intermittent running test. For the cohort, there were no differences in the heart rate (*p* = 0.071), oxygen uptake (*p* = 0.713). and lactate (*p* = 0.121) at exhaustion for the high-intensity intermittent running test.

### 3.3. High-Intensity, Intermittent Running Capacity

There was no trial order effect for the total distance covered (*p* = 0.633) and the distance covered during high-intensity running (*p* = 0.714), indicating no learning effect. Table 4 provides the cohort observations for the running distances and final running speed for the two placebo and two NZBC-extract trials at exhaustion for the high-intensity running test. For the cohort, the total distance for NZBC 1 was higher than for placebo 1 (*p* = 0.003) and placebo 2 (*p* = 0.022). NZBC 2 was higher than for placebo 1 (*p* = 0.016) with a trend for a difference between NZBC 2 and placebo 2 (*p* = 0.072). For the total distance, there was no difference between both placebo conditions (i.e., placebo 1 and 2) (*p* = 0.353) and both NZBC conditions (i.e., NZBC 1 and NZBC 2) (*p* = 0.656). Similarly, the distance covered during the high-intensity running for NZBC 1 was higher than for placebo 1 (*p* = 0.003) and placebo 2 (*p* = 0.018). NZBC 2 was higher than for placebo 1 (*p* = 0.015), with a trend for a difference between NZBC 2 and placebo 2 (*p* = 0.071). For the distance covered during the high-intensity running, there were no difference between both placebo conditions (i.e., placebo 1 and 2) (*p* = 0.254) and both NZBC conditions (i.e., NZBC 1 and NZBC 2) (*p* = 0.612).

In the NZBC extract conditions, (1) the mean total distance covered was 328 m greater (95% CI [111, 545 m], ES = 0.40, *p* = 0.006), and (2) the mean distance covered during the high-intensity running was 216 m greater (95% CI [77, 354 m], ES = 0.40, *p* = 0.005) compared to the placebo conditions. For the cohort, repeated intake of anthocyanin-rich New Zealand blackcurrant enhanced the total distance covered during the test by 7.9% (95% CI [2.0, 13.9%], range: −11.3–28.0%) and the distance covered during the high-intensity running by 8.0% (95% CI [2.1, 14.0%], range: −11.7–27.6%).

**Figure 1:**
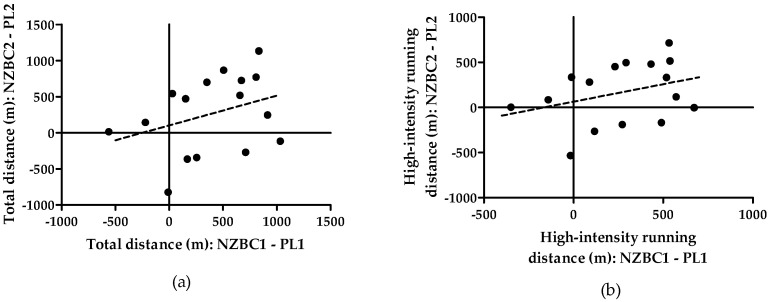
Difference between the distance in paired first and second testing session for placebo and New Zealand blackcurrant extract during the high-intensity incremental running test for (**a**) total running distance and (**b**) high-intensity running distance. NZBC, New Zealand blackcurrant; PL, placebo. Moderate correlations were not significant, indicating an inconsistent individual response for running performance with intake of New Zealand blackcurrant extract.

However, for the individual responses, the moderate correlation of 0.339 (95% CI [−0.188, 0.715], *p* = 0.198) for the two sets of placebo-adjusted NZBC extract responses for total distance covered was not significant (Figure 1a), and indicates a non-consistent individual response. In addition, the moderate correlation of 0.331 (95% CI [−0.196, 0.711], *p* = 0.209) for the two sets of placebo-adjusted NZBC extract responses for the distance covered during high-intensity running was not significant (Figure 1b), confirming also the non-consistent individual response.

In the NZBC extract condition, 8 of the 16 participants (50%) enhanced in both trials beyond the SWC for total running distance (i.e., ≥173 m) and high-intensity running distance (i.e., ≥111 m). For these repeated responders to intake of New Zealand blackcurrant, the % changes for enhanced total running distance and high-intensity running distance were 16.7% (95% CI [11.0, 22.4%], range: 7.2–28.0%) and 16.6% (95% CI [11.0, 22.2%], range: 6.7–27.6%), respectively. Three participants were considered occasional responders, with enhanced running performance in one trial beyond the SWC, and five participants could be considered non-responders.

## 4. Discussion

The primary aim of the present study was to examine the inter- and intra-individual variability in running performance response to NZBC extract, utilizing a randomized, replicated double-blinded, cross-over design. With 50% of participants demonstrating an improvement in the general magnitude of response beyond the SWC for total running distance and distance covered during high-intensity running, respectively, our observations suggest that inter-individual differences exist in response to NZBC extract, as measured by high-intensity intermittent treadmill running. In the present study, some intra-individual variability did also exist, with 19% of the participants demonstrating an improvement in total running distance and distance covered during high-intensity running beyond the SWC on one occasion, but not another.

Inter-individual variation in response to polyphenol dietary interventions is a well-recognized phenomenon [33,34]. Several intrinsic factors may influence an individual’s absorption, distribution, metabolism, and excretion (ADME) of anthocyanins, including genetic polymorphism, which may alter the expression of enzymes involved in their direct absorption, as well as phase I and II metabolism [20,35]. It has been suggested that genotype-dependent response to NZBC extract may explain some inter-individual variation [36], specifically in relation to the expression of endothelial nitric oxide synthase gene (Glu298Asp) [37]. However, pharmacogenomics studies indicate that genetic polymorphisms associated with the phase II enzyme metabolism and transport of polyphenols may also explain some inter-individual variation [34], with individuals ranging from poor to ultra-rapid metabolizers of bioactive compounds [38]. The bioavailability of anthocyanins, and their derived metabolites, has a direct link to their health and performance enhancing effects. Therefore, the inter-individual variation in ADME is likely to impact observed responses [34,35,39]. Similarly, inter-individual variations in response to sodium bicarbonate are now recognized [40], with individualized dosing of sodium bicarbonate to reduce inter-individual variation [14,41,42]. Thus, as inter-individual variation in ADME is likely to impact observed responses to NZBC extract, it is possible that the standardized supplementation strategy used may account for some inter-individual responses observed. Future research may look to establish an individualized NZBC extract supplementation strategy (timing and/or quantity) prior to examining individual responses to performance measures, such as that recently established with sodium bicarbonate [41,43].

Despite showing an improvement in HIIR capacity on one occasion (i.e., an increase > SWC) following NZBC extract supplementation, three participants (19%) did not show a general magnitude of response beyond the SWC for total distance covered or distance covered during high-intensity running. Similar inconsistencies in exercise performance have been shown in response to sodium bicarbonate, despite achieving similar levels of blood alkalosis [17]. Dias et al. [17] attributed this to the potential variation in monocarboxylate transport protein activity and the rate of H^+^ removal, thus limiting the extent to which an individual could utilize the ergogenic effects of blood alkalosis. Additionally, some intra-individual variation in response to sodium bicarbonate has been attributed to training status, with improved consistency in well-trained athletes [41]. While participants in the current study were moderately trained ($\dot{V}$O_2max_: 55.3 ± 5.0 mL·kg^−1^·min^−1^), it is possible that more highly trained individuals may demonstrate greater consistency [40,41]. In addition, research suggests the gut microbiota is relatively stable, yet its composition and activity may be modulated by factors including diet, psychological stress, and sleep deprivation [44]. While participants in the present study did not report any large deviations in diet, lifestyle, or exercise routines during the study, given the relatively long study duration (12–16 weeks) it is possible that some intra-individual variation in changes to these routines may have been attributed to some modulation of the gut microbiota. In addition, a limitation of the study is that potential confounding factors such as training load, sleep, nutritional intake outside of the trial and motivation were not recorded, and could have contributed to the variation in performance responses. However, participants were advised to maintain their habitual diets and arrive for testing well-rested. In addition, the expectations for and demands of the physical testing were discussed before the participants provided informed consent. The study had no drop-outs, potentially indicating that a lack of motivation to perform the testing can be excluded. In addition, a lack of motivation could have resulted in a trial order effect, which was not present in the study.

Despite inter- and intra-individual variations, the cohort data of the present study provides further evidence for the ergogenic potential of NZBC extract on high-intensity exercise capacity for some individuals. For the cohort, the total distance covered and high-intensity running distance increased with the intake of NZBC extract by an average of 328 m (7.9%) and 216 m (8.0%), respectively. This is in keeping with a cohort observation from a previous study, which showed an improvement of 10.8% in high-intensity running distance in the HIIR test following NZBC extract supplementation [4]. However, the present study and Perkins et al. [4] had male cohorts. Future work is needed to address whether our cohort observations in males can be replicated in a cohort study with females.

This was the first study to attempt to explore inter- and intra-individual variability in response to 7-day intake of 600 mg NZBC extract per day, utilizing a randomized, replicated, double-blind, cross-over design. However, the large variability caused by a completely randomized design [45] and the absence of repeated baseline measures meant that the data could not be fully examined using other suggested approaches to the experimental design involving two blocks with the intervention and placebo in each [20]. In the present study, our approach of a completely randomized design resulted in only six of the sixteen participants being tested in randomized blocks, as proposed by Senn [20] (see Table 1). Additionally, as two supplementation trials may not be enough to fully establish an individual as a consistent high or low responder [46], it is suggested that future research includes more baseline measures and utilizes a more traditional replicated crossover design in which sequences are more evenly matched. In addition, the dose–response effects for the intake of NZBC extract on high-intensity treadmill running performance are not known. Furthermore, it is not known whether our analysis and outcome in detecting responders using the smallest worthwhile change for a treadmill test resulted in potential meaningful changes for those responders in a competitive sport requiring high-intensity running.

When examining individual running responses, placebo trials were used to calculate SWC. It is possible that the placebo effect may have influenced running performance in these trials, influencing the final calculation of SWC. However, statistical analysis indicated no difference between the placebo trials for the total running distance or high-intensity running distance. Regardless of the method of determining response, absence of a performance effect does not imply a ‘non-response’ to NZBC extract. It is possible that individuals who did not display a response as measured by HIIR capacity, may have done so if a different measure had been used [19]. Similar results have been found when examining sodium bicarbonate supplementation, in which a mechanism for improved performance has been evident but this has not translated to enhanced exercise capacity [17]. Finally, while the treadmill protocols for participants were made relative to their _v_$\dot{V}$O_2max_ at the start of the testing schedule, it is possible that changes in their $\dot{V}$O_2max_ may have occurred during the 12–16-week period of the study, and may have altered the relative intensity of the test. It is possible that additional $\dot{V}$O_2max_ testing during and/or upon completion of the testing period would have helped to ensure the intensity remained constant; however, this additional testing may have provided an additional training stimulus.

## 5. Conclusions

It is concluded that intra-individual and inter-individual variation exist in response to NZBC extract during a high-intensity treadmill running capacity test. For 50% of the participants, seven days’ intake of NZBC extract consistently enhanced high-intensity, intermittent running capacity. In light of these data, multiple trials should be conducted to assess the intra-individual variation in response to NZBC extract before discounting or accepting its use. Likewise, multiple trials will allow for a greater understanding of an individuals’ consistency in response, prior to ergogenic supplement use. Finally, an individualized supplementation approach may be necessary to optimize the possible ergogenic effects of NZBC extract and overcome potential inter-individual variations in ADME of NZBC extract.

## Figures and Tables

**Table 1:** Trial sequence allocation. NZBC, New Zealand blackcurrant.

	Trial 1	Trial 2	Trial 3	Trial 4
Participant 1	Placebo 1	Placebo 2	NZBC 1	NZBC 2
Participant 2	NZBC 1	NZBC 2	Placebo 1	Placebo 2
Participant 3	NZBC 1	NZBC 2	Placebo 1	Placebo 2
Participant 4	NZBC 1	Placebo 1	NZBC 2	Placebo 2
Participant 5	NZBC 1	NZBC 2	Placebo 1	Placebo 2
Participant 6	Placebo 1	Placebo 2	NZBC 1	NZBC 2
Participant 7	NZBC 1	NZBC 2	Placebo 1	Placebo 2
Participant 8	NZBC 1	NZBC 2	Placebo 1	Placebo 2
Participant 9	Placebo 1	NZBC 1	NZBC 2	Placebo 2
Participant 10	Placebo 1	NZBC 1	NZBC 2	Placebo 2
Participant 11	Placebo 1	Placebo 2	NZBC 1	NZBC 2
Participant 12	Placebo 1	Placebo 2	NZBC 1	NZBC 2
participant 13	NZBC 1	NZBC 2	Placebo 1	Placebo 2
Participant 14	Placebo 1	NZBC 1	NZBC 2	Placebo 2
Participant 15	NZBC 1	Placebo 1	Placebo 2	NZBC 2
Participant 16	Placebo 1	NZBC 1	Placebo 2	NZBC 2

**Table 2:** Heart rate, oxygen uptake and lactate for stage 3 of the high-intensity intermittent running test. NZBC, New Zealand blackcurrant; $\dot{V}$O_2_, oxygen uptake.

	Placebo 1	Placebo 2	NZBC 1	NZBC 2
Heart rate	170 ± 16	171 ± 13	171 ± 13	171 ± 12
95% CI heart rate	[161, 179]	[164, 178]	[164, 178]	[165, 178]
(beats·min^−1^)				
$\dot{V}$O_2_	43.5 ± 3.1	43.4 ± 4.3	43.4 ± 3.1	43.7 ± 3.4
$95\%\;\mathrm{CI}\;\dot{V}$O_2_	[41.7, 45.3]	[40.9, 45.9]	[41.6, 45.2]	[41.7, 45.71]
(mL·kg^−1^·min^−1^)				
Lactate	2.88 ± 0.91	3.14 ± 0.90	3.09 ± 0.88	3.27 ± 0.97
95% CI lactate	[2.40, 3.36]	[2.66, 3.61]	[2.63, 3.56]	[2.76, 3.79]
(mmol·L^−1^)				

**Table 3:** Heart rate, oxygen uptake and lactate at exhaustion during the high-intensity intermittent running test. NZBC, New Zealand blackcurrant; $\dot{V}$O_2_, oxygen uptake.

	Placebo 1	Placebo 2	NZBC 1	NZBC 2
Heart rate	191 ± 10	192 ± 9	194 ± 8	195 ± 7
95% CI heart rate	[185, 196]	[187, 196]	[190, 198]	[192, 199]
(beats·min^−1^)				
$\dot{V}$O_2_	54.0 ± 5.4	53.8 ± 6.4	54.0 ± 5.9	54.6 ± 6.1
$95\%\;\mathrm{CI}\;\dot{V}$O_2_	[50.9, 57.1]	[50.1, 57.5]	[50.6, 57.4]	[51.1, 58.1]
(mL·kg^−1^·min^−1^)				
Lactate	4.84 ± 1.19	5.49 ± 1.91	5.47 ± 1.47	5.46 ± 1.17
95% CI lactate	[4.20, 5.47]	[4.47, 6.51]	[4.68, 6.25]	[4.84, 6.08]
(mmol·L^−1^)				

**Table 4:** Running distances and final running speed for each trial (mean ± SD). TRD, total running distance; HIRD, high-intensity running distance; NZBC, New Zealand blackcurrant.

	Placebo 1	Placebo 2	NZBC 1	NZBC 2
TRD (m)	3741 ± 788	3818 ± 957	4135 ± 934	4080 ± 677
95% CI TRD (m)	3321, 4161	3308, 4327	3637, 4632	3719, 4441
HIRD (m)	2386 ± 510	2444 ± 608	2651 ± 617	2610 ± 451
95% CI HIRD (m)	2114, 2657	2120, 2768	2322, 2979	2370, 2851
Final running	17.6 ± 1.5	17.8 ± 1.6	18.2 ± 1.4	18.2 ± 1.1
speed (km·h^−1^)				

## Data Availability

Data are available on reasonable request.
